# Short Communication: Preliminary Differences Identified in Genes Responsible for Biofilm Formation in Poultry Isolates of *Salmonella enterica* Heidelberg, Enteritidis, and Kentucky

**DOI:** 10.3390/microorganisms7070196

**Published:** 2019-07-09

**Authors:** Zhaohao Shi, Dana K. Dittoe, Kristina M. Feye, Mike Kogut, Steven C. Ricke

**Affiliations:** 1 Center for Food Safety and Food Science Department, University of Arkansas, Fayetteville, AR 72704, USA; 2 Southern Plains Agricultural Research Center, United States Department of Agriculture-Agricultural Research Unit, College Station, TX 77845, USA

**Keywords:** biofilm, pellicle, *Salmonella* Enteritidis, *Salmonella* Kentucky, *Salmonella* Heidelberg, qRT-PCR

## Abstract

*Salmonella enterica* is one of the most prevalent foodborne pathogens. The large quantity of serovar types results in the colonization of a large spectrum of hosts, with different environmental conditions and hazards. The aim of this study was to evaluate the differences in gene expression (*bcsA* and *csgD*) of *Salmonella enterica* serovars Heidelberg, Kentucky, and Enteritidis during biofilm formation using quantitative reverse-transcriptase polymerase chain reaction (qRT-PCR). Overall, there appeared to be differences in expression between the different serovars with high variation between strains. These data are important as they demonstrate considerable variability in gene expression between serovars and strains of poultry isolates of *Salmonella enterica*.

## 1. Introduction

Non-typhoidal *Salmonella enterica* is one of the most commonly encountered bacterial foodborne pathogens, with over 2500 serovars currently documented with several associated with produce, meat, and poultry products [1,2,3,4,5,6]. *Salmonella* serovars Enteritidis, Heidelberg, and Kentucky are among the five most identified serovars from poultry and poultry products [2,4,5,7,8]. *Salmonella* Enteritidis is considered the most frequently associated with the consumption of undercooked poultry and egg products [9,10]. *Salmonella* Heidelberg is also associated with eggs. However, it is more typically identified with outbreaks of contaminated poultry meat [11,12,13,14]. While *Salmonella* Kentucky is commonly found, it is uncommonly associated with foodborne disease [7]. Therefore, it is important to investigate the differences between host-adapted serovars that exhibit an altered disease potential in different hosts, such as *Salmonella* Enteritidis, versus host-restricted serovars that cause significant pathology in closely related host species [15,16,17,18]. While *Salmonella* Kentucky is a rare foodborne disease, the ability to survive in the environment and persist is intriguing.

Outside of the host, *Salmonella* expresses a variety of survival mechanisms to the environment, one of which is the ability to form biofilms. Biofilms enable *Salmonella* to resist antimicrobials and thrive in a variety of habitats [19]. Biofilms may form on biotic surfaces, such as on organic plant structures, or abiotic surfaces, such as stainless steel and plastic. Biofilms can also form what is termed a pellicle at the air-liquid interface [20]. Because of this ability, *Salmonella* can colonize a food processing area and form a biofilm. Any *Salmonella* strains allowed to persist may form stronger biofilms in the future and become more resistant to removal than freshly introduced strains [21]. This process is enhanced with the development of the biopolymer matrix of extracellular polymeric substances (EPS) within the pellicle.

Comprising up to 90% of the total matter of a biofilm, EPS plays a pivotal role in functions related to structural support, nutrient transport, and protection [22]. In *Salmonella*, the principle polysaccharide structural component of the EPS matrices is cellulose, chiefly regulated by the *bcs* (bacterial cellulose synthase) operon, without which, *Salmonella* is unable to form strong biofilms [23]. The other major component of the matrix is the amyloid proteinaceous curli fimbriae structures controlled by the *csg* operons (curli specific gene) which interact with cellulose to start the formation of biofilms and enhance the sequential survival [24,25]. These two structural components provide the majority of the EPS for *Salmonella* and are critical in the ability to form a biofilm.

The objective of the current study was to examine the differences in the expression of critical *Salmonella* biofilm structural genes across several serovars. *Salmonella* Enteritidis and Heidelberg are important foodborne pathogens, whereas Kentucky is pervasive yet mostly benign and serves as an interesting juxtaposition. Expression levels of the *bcsA* and *csgD* genes were observed in strains of *Salmonella* serovars Enteritidis, Heidelberg, and Kentucky over the course of the development of bacterial pellicles across a 96-h time period. The authors hypothesized that the different serovars would exhibit contrasting expression levels of the two genes as assessed using quantitative reverse-transcriptase polymerase chain reaction assays (qRT-PCR).

## 2. Materials and Methods

### 2.1. Bacterial Strains and Pellicle Formation

Three serovars of *Salmonella* consisting of three unique strains from the University of Arkansas Center for Food Safety Culture Collection were used in this study. This included three strains of *S.* Kentucky (UA CFS# 38-0055, 38-0084, 38-0085), three strains of *S.* Enteritidis (UA CFS# 38-0086, 38-0087, 38-0088), and three strains of *S.* Heidelberg (UA CFS# 38-00126, 38-00127, 38-00128). Quadrant streaks of the isolates from frozen stocks were prepared on Luria–Bertani (LB) (BD Biosciences, Franklin Lakes, NJ, USA) agar plates and incubated for 24 h at 37 °C. After incubation, single colonies were selected and grown in 5 mL of LB broth overnight in a 37 °C shaking incubator for 18 h. Overnight cultures of each of the *Salmonella* strains were diluted 1:10 and inoculated into 125 mL flasks containing 50 mL of LB broth without salt (10 g tryptone and 5 g yeast extract per L) (BD Biosciences, Franklin Lakes, NJ, USA). Flasks were placed at room temperature for 96 h with the planktonic cultures developing pellicles by the end of this period. At the 0 h, 24 h, 48 h, 72 h, and 96 h time points, 1 mL of culture was collected directly below the meniscus and total RNA was extracted using a Qiagen RNeasy kit (Qiagen, Valencia, CA, USA). RNA was stored at −80 °C until qRT-PCR was performed. Two independent trials were performed for this study.

### 2.2. Quantitative Reverse-Transcriptase PCR (qRT-PCR) Assay

The qRT-PCR assays were performed using the Verso 1-Step RT-qPCR Kit (Thermo Scientific, Waltham, MA, USA) and optimized using an Eppendorf RealPlex^TM^ Mastercycler Epigradient Thermocycler (Eppendorf, Hamburg, Germany). To remove any DNA remaining after RNA extraction, the RNA samples were treated with DNase I (Invitrogen, Carlsbad, CA, USA) before each assay. Primer pairs for the *csgD, bcsA,* and the rRNA housekeeping gene *rsmC* were synthesized by Integrated DNA Technologies (IDT, Coralville, IA, USA) and confirmed using the National Center for Biotechnology Information (NCBI) BLAST. Primer sets yielded amplicons that were 156, 136, and 190 bp for the putative *bcsA*, *csgD*, and *rsmC* genes. A mastermix was prepared to ensure that each 25 µL reaction contained the following: 12.5 µL of 2× 1-Step qPCR SYBR Mix (Thermo Scientific, Waltham, MA, USA), 1.25 µL RT Enhancer, 0.25 µL Verso Enzyme mix, 500 nM of each forward and reverse primer, 100 ng of total RNA template, and nuclease-free water (MBI Growcells, Irving, CA, USA). The qRT-PCR conditions consisted of a 5-minute cDNA synthesis step 50 °C step followed by a 15 m Hot Start at 95 °C. Then, 40 cycles consisting of 15 s for denaturation at 95 °C, 15 s for primer annealing at 55 °C, and 20 s for amplicon extension at 68 °C was performed with melt curves. The melt curves were produced by cycling form of 95 °C for 15 s and then by 60 °C for 20 m with a 0.5 °C increase in temperature per minute until a final temperature of 95 °C was reached. Each assay was performed in triplicate.

### 2.3. Statistical Analysis

In order to study the differences in gene expression among the three *Salmonella* serovars, we observed the RNA transcript levels of the pellicle structural genes *csgD* and *bcsA* as determined by qRT-PCR. The CT value was taken from 40, which is the maximum number of cycles, to give the relative abundance of CT (nCT). Therefore, an increase in CT means there is an increase in gene expression, a decrease in CT means there is a decrease in gene expression. The differences in CT values were compared with values exhibited by the rRNA housekeeping gene *rsmC* and analyzed using the JMP^®^ 14.0 (SAS Institute, Cary, NC, USA) software suite. Data were analyzed using n-way ANOVA. Means were separated using Tukey’s protected HSD with a significance level of *p* ≤ 0.05. The SEM, or standard error of measurement, was included as it represents the variation of the data across the average.

## 3. Results

### 3.1. CsgD Expression

**Figure 1:**
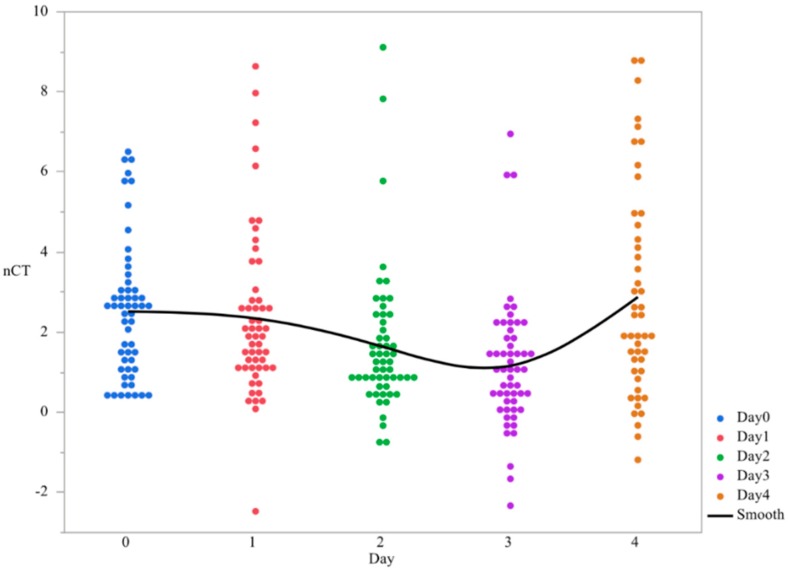
Changes in expression of the gene *csgD* from planktonic cells used to form *Salmonella* pellicles over a 4-days time period ^1^. qRT-PCR was performed on total RNA isolated from bacterial cultures of *Salmonella* as pellicles were being formed over a 4-day time period. ^1^ N = 270, *n* = 54, *p* < 0.0001, Individual SEM for day 0, 1, 2, 3, and 4 was 0.231, 0.285, 0.242, 0.226, and 0.387 respectively.

**Figure 2:**
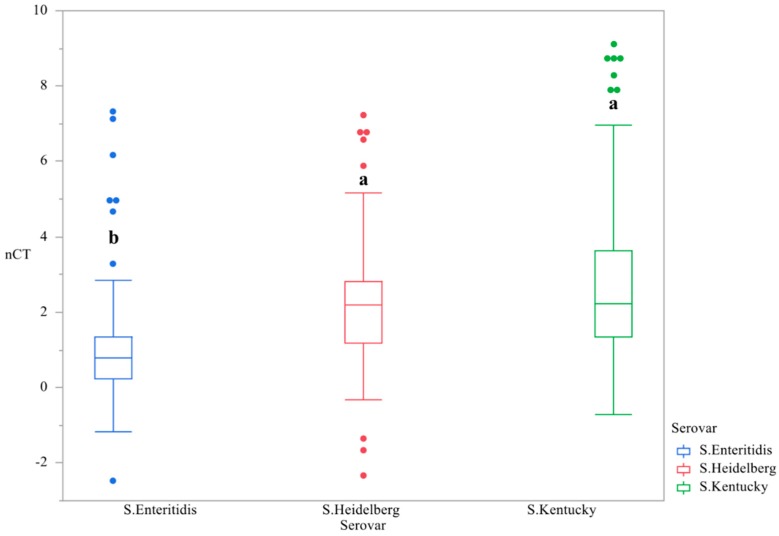
Differences in expression of the gene *csgD* between serovars ^1,2^. qRT-PCR was performed on total RNA isolated from bacterial cultures of *Salmonella* as pellicles were being formed over a 4-day time period. ^1^ N = 270, *n* = 90, *p* < 0.0001, Individual SEM was 0.170, 0.184, 0.253 for *S.* Enteritidis, Heidelberg, and Kentucky. ^2^ Means with different superscripts are considered significantly different (a–b).

First, the main effect of time was evaluated in order to determine if changes in gene expression were temporal. The temporal variation of c*sgD* expression throughout the study was demonstrated, with the peak of gene expression at day 3. (*p* < 0.0001, Figure 1, Table 1). The main effect of serovar was significant, with *S.* Kentucky and Heidelberg exhibiting lower levels of gene expression than *S.* Enteritidis (*p* < 0.0001, Figure 2). The gene expression for day 4 was the most divergent (*p* < 0.05, Table 2).

**Figure 3:**
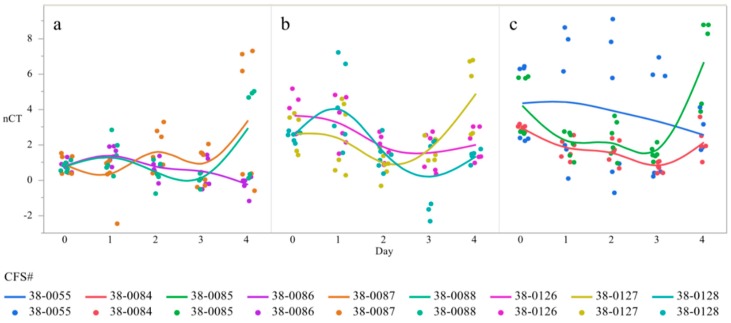
Differences in expression of the gene *csgD* over time in strains of *Salmonella* Heidelberg (**a**), Enteritidis (**b**),and Kentucky (**c**) ^1^. qRT-PCR was performed on total RNA isolated from bacterial cultures of *Salmonella* as pellicles were being formed over a 4-day time period ^1^ N = 90, *n* = 6, *p* = 0.0006 (**a**), 0.003 (**b**), 0.0211 (**c**).

In order to determine if the changes in gene expression between serovars were driven by specific strains, poultry isolates of *Salmonella* Heidelberg, (38-0055, 38-0084, 38-0085), Enteritidis (38-0086, 38-0087, 38-0088) and Kentucky (38-0126, 38-0127, 38-0128) over time were evaluated. The interaction between strain and serovar was statistically significant, with a demonstrated variation between strains within serovar (Table 2). *Salmonella* Kentucky 38-0085 showed the lowest level of gene expression on day 4 compared to 38-0055 and 38-0084 (Table 2). Two of the three strains increased their gene expression on day 1, with all strains decreasing gene expression relative to day 3 on day 4. Additionally, *S*. Kentucky strain 38-0084 exhibited a higher magnitude of gene expression changes throughout the study as compared to other strains (Table 2). *S*. Heidelberg expressed less variation, with day 2 relative to day 1 and day 4 relative to day 1 exhibiting the same direction of variation, though the magnitude was different. Finally, *S*. Enteritidis demonstrated the highest level of gene expression and the most dramatic variation in the interaction between strain and time (Table 2). Therefore, there are significant interactions in the differences in *csgD* expression between the serovars, as well as the strains independently, across time. Temporal expression of csgD was polynomial, with gene expression increasing from day 0 to day 1, dropping by day 3, and then increasing by day 4 (Figure 3).

### 3.2. BcsA Expression

**Figure 4:**
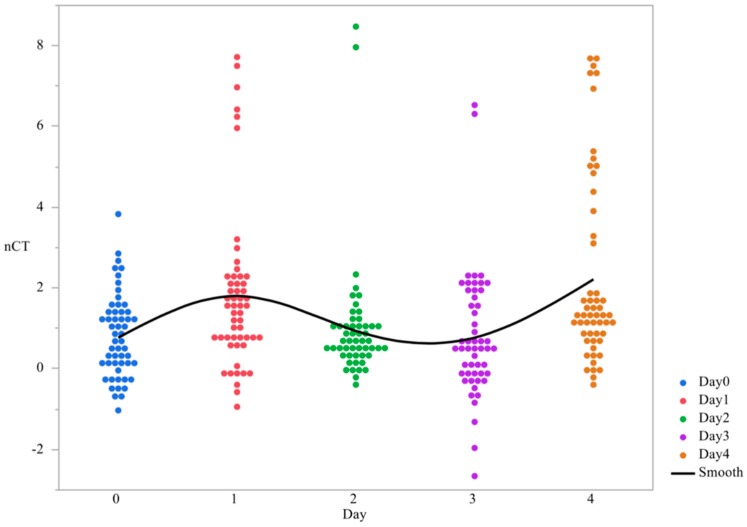
Changes in expression of the gene *bcsA* from planktonic cells used to form *Salmonella* pellicles over a 4-days time period ^1^. qRT-PCR was performed on total RNA isolated from bacterial cultures of *Salmonella* as pellicles were being formed over a 4-day time period. ^1^ N = 270, *n* = 54, *p* < 0.0001, Individual SEM for day 0, 1, 2, 3, and 4 was 0.138, 0.273, 0.219, 0.220, and 0.319 respectively.

**Figure 5:**
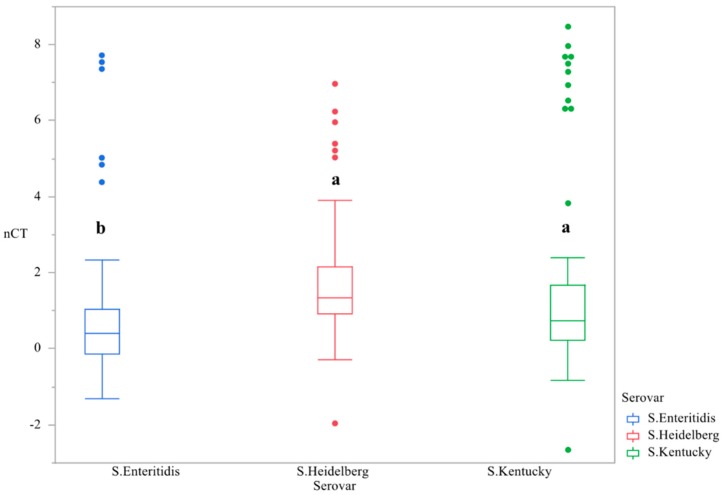
Differences in expression of the gene *bcsA* between serovars ^1,2^. qRT-PCR was performed on total RNA isolated from bacterial cultures of *Salmonella* as pellicles were being formed over a 4-day time period. ^1^ N = 270, *n* = 90, *p* < 0.0019, Individual SEM was 0.175, 0.152, 0.242 for *S.* Enteritidis, Heidelberg, and Kentucky. ^2^ Means with different superscripts are considered significantly different (a–b).

In order to evaluate the differences in the overall gene expression across time irrespective of serovar, the main effect of time for *bcsA* expression was evaluated. The overall trend of the expression was statistically significant and was highly variable. Overall expression levels of *bcsA* showed polynomial trends in temporal expression, with gene expression increasing from day 0 to day 1, followed by a decrease on day 2 and day 3, and then increase again on day 4 (*p* < 0.0001, Table 3, Figure 4). The greatest difference in gene expression differences of *bcsA* was between day 0 and day 4, with a 2.89-fold change reduction in gene expression to day 0 (Table 2). When comparing the main effect of serovars, *S*. Enteritidis and *S*. Kentucky demonstrated the highest gene expression overall (*p* = 0.0019, Figure 5).

**Figure 6:**
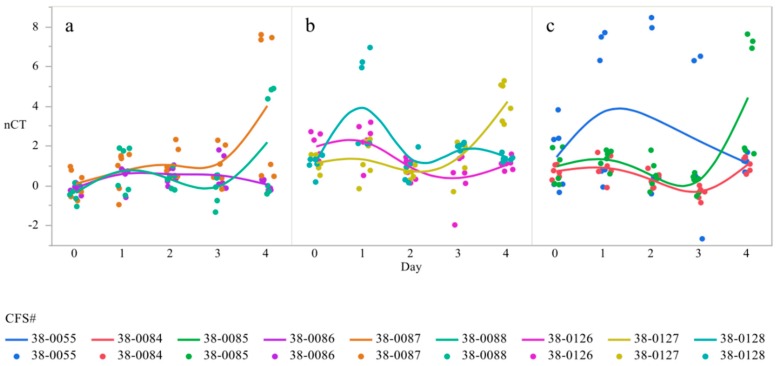
Differences in expression of the gene *bcsA* over time in strains of *Salmonella* Heidelberg (**a**), Enteritidis (**b**),and Kentucky (**c**) ^1^. qRT-PCR was performed on total RNA isolated from bacterial cultures of *Salmonella* as pellicles were being formed over a 4-day time period ^1^ N = 90, *n* = 6, *p* < 0.0001 (**a**), 0.0222 (**b**), 0.0152 (**c**).

The interaction between strain and time was also significant, though it was not as dynamic as the gene expression differences of *csgD* (*p* < 0.05, Table 4). *S.* Kentucky strain 38-0084 showed the greatest gene expression difference between day 2 and day 3 as compared to the other serovars (*p* = 0.0152, Figure 6c). Additionally, *S.* Kentucky strain 38-0085 demonstrating the lowest gene expression on day 4 as compared to the other strains (*p* = 0.0152, Figure 6c). Unlike the two other serovars, *S.* Enteritidis strains were more or less volatile in their expression as compared to the other serovars. There was some variation on gene expression on day 4, with strain 38-087 and 38-088 *bcsA* expression being the most divergent from 38-086 (*p* < 0.0001, Figure 6a). *S.* Heidelberg was slightly more dynamic than the other two serovars (*p* = 0.0222, Figure 6b). On day 1, 38-0127 and 38-1026 expressed more *bcsA* as compared to 38-1028 (Figure 6b). Strain 38-1026 had higher levels of gene expression as compared to the other strains on day 3 (Figure 6b). Finally, day 4 *bcsA* expression was the lowest in 38-0127. Therefore, while the strains were not as volatile in their gene expression of *bcsA* and *csgD*, differences between strain and serovar existed.

### 3.3. Ratio of csgD to bcsA Expression

**Figure 7:**
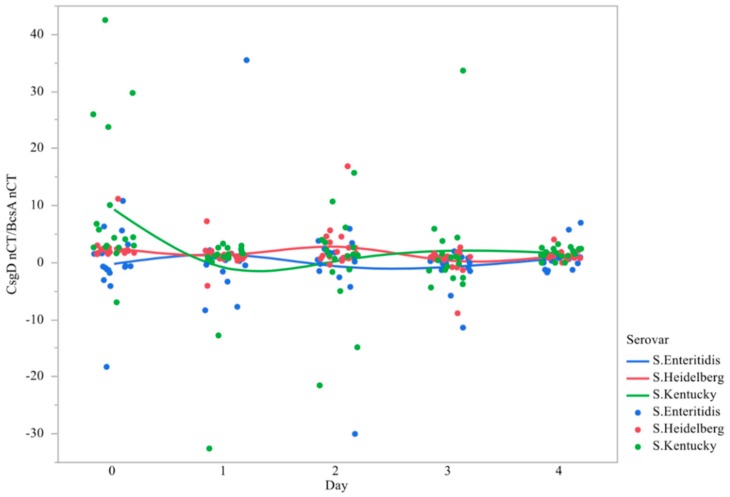
Comparison of changes in expression of the ratio of the genes *csgD* and *bcsA* in *Salmonella* serovars over time 1. qRT-PCR was performed on total RNA isolated from bacterial cultures of *Salmonella* as pellicles were being formed over a 96 h time period. Individual SEM was 1.450, 2.126, 1.808, 0.741, and 0.560, for *S.* Enteritidis, 0.516,0.472, 0.963, 0.620, and 0.209 for *S.* Heidelberg, and 2.960, 2.026, 2.062, 2.067, and 0.227 for *S.* Kentucky on day 0, 1, 2, 3, and 4, respectively. ^1^ N = 270, *n* = 18, *p* =0.0065.

In order to compare the differences in gene expression magnitude between *csgD* versus *bcsA*, the ratio of the nCTs were taken. It should be noted that an upregulation of *csgD* initiates the *bcsA* operon. If their ratio was similar across all serovars and time, then mechanistically it is theorized that the transcriptional mechanism guiding cellulose pellicle formation is conserved between serovars. Data indicate that there is significant variation in fold change *csgD/bcsA* between serovars, although there is no significant difference in the interaction between strain and time (Table 5, Figure 7). We theorize that the differences in gene expression between these two genes are temporally unique, and therefore, may be mechanistically different. A greater number of replications across numerous strains would likely prove that point.

Starting at day 0 *Salmonella* Heidelberg and Kentucky, *csgD*/*bcsA* fold change was greater than Enteritidis, meaning *csgD* expression was higher prior to pellicle formation. By day 1, the difference in the ratio of *csgD*/*bcsA* was not different between serovars. The ratio increases significantly by day 3 for Enteritidis and Heidelberg and decreases for Kentucky, meaning Kentucky expresses less *csgD* than the other two serovars. All serovars had similar ratios in their gene expression on day 4.

## 4. Discussion and Conclusions

Extracellular biofilm formation in *Salmonella* requires polysaccharide cellulose and curli amyloid fimbrial structures [24]. Together, these biopolymers provide structure to the biofilm and support cell adhesion with the presence of both being necessary to produce fully functional and maximally resistant biofilms [23]. The transcriptional regulator *csgD* controls the production of curli fimbriae by positively regulating the *csgBA* operon which produces the protein components of curli [26]. In addition, *csgD* acts on the *adrA* promoter section, which results in the production of cyclic diguanylic acid (c-di-GMP), an allosteric activator of cellulose synthase encoded by the *bcs* operon [27]. Therefore, the activation of *csgD* not only produces curli proteins, but activates the *bcsA* operon. Because of the critical role these two genes provide in *Salmonella* biofilm formation, they were chosen for this study. Because the temporal activation of this machinery seemed to be different between serovars, as evidence from our study indicates, there may be serovar-specific mechanisms of biofilm formation. Whether this means that biofilms form sooner or later or that the machinery that drives the formation of the biofilm is executed differently, this could explain differences in recoverable *Salmonella* from biofilms in the poultry industry. The potential difference between serovars may also be exploitable. More evidence is needed to determine the full impact of our findings, including transcriptomic and proteomic studies evaluating biofilm formation differences between serovars. For instance, if Kentucky somehow does not produce biofilms as rapidly, maybe it is cleared better in specific environmental or physiological conditions or it is more susceptible to antimicrobials.

As such, it would be expected that increases in *csgD* expression would lead to increases in cellulose production and require the expression of the *bcsA* gene. In our current preliminary experiment, we found that each serovar exhibited a unique pattern of gene expression across time, with *csgD* a being much more volatile than *bcsA*. Furthermore, the changes of expression varied considerably over the days between all of the individual strains within serovar. Considering the ratio of *csgD* to *bcsA*, there were also minor differences between each serovar. Despite differences in the magnitude and direction of gene expression between different strains of *Salmonella*, the ratios were not significantly different in this pilot study. Additional data are required, including more strains, more replications, and additional gene expression analyses. By expanding on this study, we may potentially find differences between serovars that can serve as targets in the future for the development of new antimicrobials. Additionally, if one serovar expresses cellulose greater than the other, that particular gene cassette may be used for the production of cellulose.

Our study found there to be differences in the expression of biofilm-forming genes between serovars and large variations in gene expression between strains within each serovar. Future assessments should include a broader variety of serovars and additional replications to validate the qPCR data found in this paper. The replication and expansion of this data set must include additional common poultry isolates that are important to human health. However, the data presented herein are promising because if proven true, could potentially lead to novel understandings of individual serovar gene expression differences in biofilm formation.

## Figures and Tables

**Table 1:** Changes in expression of the gene CsgD from planktonic cells used to form Salmonella pellicles over a 4-day time period ^1,2^.

	Day				
	0	1	2	3	4
*CsgD* nCT	2.157 ± 0.231 ^ab^	2.346 ± 0.285 ^ab^	1.641 ± 0.242 ^bc^	1.151 ± 0.226 ^c^	2.884 ± 0.387 ^a^

^1^ N = 270, *n* = 54, Means of nCT values and individual SEMs are given for each strain at each time point. ^2^ Means with different superscripts are considered significantly different (a–c).

**Table 2:** Changes in expression of the gene *csgD* from planktonic cells used to form pellicles in *Salmonella* Kentucky, Enteritidis, and Heidelberg over a 4-day time period.^1,2,3^.

	Day				
	0	1	2	3	4
*S*. Enteritidis					
38-0086	0.788 ± 0.139 ^abc^	1.442 ± 0.190 ^abc^	0.667 ± 0.21 ^bc^	0.53 ± 0.385 ^bc^	−0.28 ± 0.24 ^c^
38-0087	0.887 ± 0.227 ^abc^	0.218 ± 0.553 ^bc^	1.843 ± 0.472 ^abc^	0.722 ± 0.437 ^abc^	3.449 ± 1.545 ^a^
38-0088	0.762 ± 0.108 ^abc^	1.328 ± 0.389 ^abc^	0.438 ± 0.284 ^bc^	0.057 ± 0.141 ^c^	3.008 ± 1.142 ^ab^
*S*. Heidelberg					
38-0126	3.617 ± 0.461 ^abc^	3.267 ± 0.565 ^abcd^	1.767 ± 0.317 ^bcde^	1.552 ± 0.451 ^bcde^	2.002 ± 0.374 ^bcde^
38-0127	2.575 ± 0.452 ^abcde^	2.43 ± 0.81 ^abcde^	0.86 ± 0.305 ^de^	1.794 ± 0.261 ^bcde^	4.928 ± 0.956 ^a^
38-0128	2.483 ± 0.111 ^abcde^	4.182 ± 0.928 ^ab^	1.363 ± 0.343 ^cde^	0.155 ± 0.876 ^e^	1.383 ± 0.152 ^bcde^
*S*. Kentucky					
38-0055	4.333 ± 0.904 ^ab^	4.425 ± 1.476 ^ab^	3.898 ± 1.714 ^ab^	3.313 ± 1.33 ^ab^	2.538 ± 0.474 ^ab^
38-0084	2.955 ± 0.068 ^ab^	1.752 ± 0.235 ^b^	1.628 ± 0.263 ^b^	0.719 ± 0.117 ^b^	2.186 ± 0.421 ^b^
38-0085	4.256 ± 0.692 ^ab^	2.068 ± 0.296 ^b^	2.308 ± 0.427 ^b^	1.522 ± 0.155 ^b^	6.809 ± 1.114 ^a^

^1^ Each serovar was analyzed separately from others to determine the interaction between specific strains within strains and day. Means of nCT values and individual SEMs are given for each strain at each time point. ^2^ N = 90, *n* = 6. ^3^ Means with different superscripts in the same serovar are considered significantly different with serovar (a–e).

**Table 3:** Changes in expression of the gene *bcsA* from planktonic cells used to form *Salmonella* pellicles over a 4-day time period ^1,2^.

	Day				
	0	1	2	3	4
*BcsA* nCT	0.766 ± 0.138 ^c^	1.815 ± 0.273 ^ab^	0.932 ± 0.219 ^bc^	0.766 ± 0.22 ^c^	2.217 ± 0.319 ^a^

^1^ N = 270, *n* = 54, Means of nCT values and individual SEMs are given for each strain at each time point. ^2^ Means with different superscripts are considered significantly different (a–c).

**Table 4:** Changes in expression of the gene *bcsA* from planktonic cells used to form pellicles in *Salmonella* Kentucky, Enteritidis, and Heidelberg over a 4-day time period ^1,2,3^.

	Day				
	0	1	2	3	4
*S*. Enteritidis					
38-0086	−0.115 ± 0.101 ^b^	0.645 ± 0.275 ^b^	0.557 ± 0.175 ^b^	0.558 ± 0.354 ^b^	0.059 ± 0.105 ^b^
38-0087	0.107 ± 0.296 ^b^	0.742 ± 0.423 ^b^	1.14 ± 0.315 ^b^	1.022 ± 0.402 ^b^	4.089 ± 1.521 ^a^
38-0088	−0.372 ± 0.175 ^b^	0.823 ± 0.467 ^b^	0.343 ± 0.184 ^b^	−0.103 ± 0.314 ^b^	2.247 ± 1.105 ^ab^
*S*. Heidelberg					
38-0126	1.948 ± 0.284 ^bc^	2.313 ± 0.39 ^b^	0.852 ± 0.219 ^bc^	0.392 ± 0.513 ^c^	1.097 ± 0.124 ^bc^
38-0127	1.133 ± 0.162 ^bc^	1.39 ± 0.402 ^bc^	0.702 ± 0.118 ^bc^	1.433 ± 0.39 ^bc^	4.281 ± 0.4 ^a^
38-0128	1.098 ± 0.194 ^bc^	4.263 ± 0.961 ^a^	0.899 ± 0.321 ^bc^	2.017 ± 0.071 ^bc^	1.39 ± 0.068 ^bc^
*S*. Kentucky					
38-0055	1.403 ± 0.688 ^abc^	3.842 ± 1.511 ^ab^	3.382 ± 1.982 ^abc^	2.228 ± 1.805 ^abc^	1.134 ± 0.159 ^abc^
38-0084	0.727 ± 0.128 ^abc^	0.938 ± 0.26 ^abc^	0.332 ± 0.16 ^bc^	−0.359 ± 0.126 ^c^	1.126 ± 0.149 ^abc^
38-0085	0.963 ± 0.361 ^abc^	1.378 ± 0.183 ^abc^	0.587 ± 0.302 ^abc^	0.16 ± 0.192 ^bc^	4.532 ± 1.237 ^a^

^1^ Each serovar was analyzed separately from others to determine the interaction between specific strains within strains and day. Means of nCT values and individual SEMs are given for each strain at each time point. ^2^ N = 90, *n* = 6. ^3^ Means with different superscripts in the same serovar are considered significantly different with serovar (a–e).

**Table 5:** Comparison of changes in expression of the ratio of the genes *csgD* and *bcsA* in the interaction between *Salmonella* serovars and a 4-day time period ^1,2^.

	Day				
	0	1	2	3	4
*S.* Enteritidis	−0.230 ± 1.450 ^b^	1.485 ± 2.126 ^b^	−0.696 ± 1.808 ^b^	0.741 ± 0.741 ^b^	0.768 ± 0.530 ^b^
*S.* Heidelberg	2.575 ± 0.516 ^ab^	1.235 ± 0.472 ^b^	2.936 ± 0.963 ^ab^	0.2638 ± 0.620 ^b^	1.384 ± 0.205 ^b^
*S.* Kentucky	9.400 ± 2.960 ^a^	−1.083 ± 2.026 ^b^	0.406 ± 2.062 ^b^	2.101 ± 2.087 ^ab^	1.646 ± 0.227 ^b^

^1^ N = 270, *n* = 18, *p* = 0.0065, Means of nCT values and individual SEMs are given for each strain at each time point. ^2^ Means with different superscripts are considered significantly different (a–b).
